# Viewpoint: Toward Involvement of Caregivers in Suicide Prevention Strategies; Ethical Issues and Perspectives

**DOI:** 10.3389/fpsyg.2018.02457

**Published:** 2018-12-18

**Authors:** Valérie Le Moal, Christophe Lemey, Michel Walter, Sofian Berrouiguet

**Affiliations:** ^1^Brest Medical University Hospital at Bohars, Adult Psychiatry, Bohars, France; ^2^IMT Atlantique, Lab-STICC, Brest, France

**Keywords:** suicide, attempted-caregiver, designated-ethics-responsibility-vulnerability, prevention strategies, ethical issues

## AIM

The aim of this article is to investigate the potential impacts implication of caregivers as a resource in care management of patients at risk of suicide.

## Suicide Prevention Challenges

Suicide prevention research faces specific challenges related to characteristics of suicide attempts and attempters (Wasserman, 2004). Suicide attempters have been described as poorly adhering to intensive treatment over time, and delivery of interventions in the emergency department can be difficult, where psychiatric staff availability is often limited or absent. The post-discharge period constitutes a critical challenge for emergency and mental health care services both in the short and long terms (Hunt et al., 2009). Given these issues, there has been growing interest in assessing the efficacy of post discharge intervention after a suicide attempt. For example, brief contact interventions (BCIs) are low resources interventions seeking to maintain long-term contact with patients after a suicide attempt (Milner et al., 2015). These interventions intent to reinforce the health care networking around the patient but only rely on mental health and emergency services. Depression, drug misuse, family and social situations are well established suicide risk factors (Zalsman et al., 2016). Recent findings also showed the correlation between sleep disturbances and suicidality from a clinical point of view (Pompili et al., 2013). Including protective factors in suicide risk management might be of great interest. However, prevention strategies often exclude form the preventative procedure an essential preventative component of patients social network: the caregiver (Mann et al., 2005). Indeed, studies showed the potential positive impacts of caregivers in the management of important suicide risk factors as depression (Joling et al., 2012), and social isolation (Chatterjee et al., 2014).

## Involving the Caregivers in the Suicide Prevention Process

A caregiver, or carer, is an unpaid or paid member of a person's social network who helps them with activities of daily living. Caregiving is most commonly used to address impairments related to old age, disability, a disease, or a mental disorder (Berk et al., 2013).

Referred to as informal caregivers, caregivers provide a complex array of support tasks that extend across physical, psychological, spiritual, and emotional domains. Studies have shown that caregivers and close contacts are reliable sources of information about patients with psychiatric disorders. Caregivers do not exactly provide medical care, but can be considered as partners in the care as suggested by Fredman and Daly (Fredman and Daly, 1993). Traditional psychiatric assessment, however, does not always include information from caregivers due to time constraints, concerns about confidentiality and the risk of caregiver burden (Adelman et al., 2014). By excluding caregivers from assessments, clinicians may miss an opportunity to obtain additional valuable information about the illness course. Including caregivers in innovative prevention strategies could strongly improve the insight regarding patients' suicide risk situation.

Moreover, the involvement of the caregivers in a patient's assessment may facilitate the implementation of a step-by-step personalized prevention program during care transitions (e.g., hospital to home). This strategy may evolve into an all-embracing, tailored partnership involving healthcare professionals, patients, and their caregiver. Furthermore, the involvement of the caregivers means that they are able to play an important part in providing support and detecting warning signs when indicated. Caregivers are potential allies in the suicide prevention without, however, taking the place of healthcare professionals (Sun and Long, 2008).

## Caregiver Involvement: Opportunities and Issues

Despite these promising opportunities, issues are raised regarding the assimilation of caregivers designated by patients at risk of suicide. Support for the patient on the part of his/her network in the vast majority of cases creates opportunities to relieve relatives of the strain of overwork. However, some studies have intended to involve the caregiver in a suicide prevention approach. For example, according to Sun et al., caregivers are able to play an important part in providing support and detecting warning signs and are potential allies in suicide prevention (Sun et al., 2009).

Family caregivers' suicide caring competence is important to prevent their relatives with suicidal tendencies from attempting suicide. Authors stated that clinicians and nurses are typically educated and trained to care for patients with suicidal tendencies, but family caregivers of suicidal individuals do not receive the same level of suicide care education. Family caregivers may lack competence to care for their relatives with suicidal ideations and/or behaviors. In this perspective, Sun et al. proposed an assessment of suicide caring competence which may help clinicians to assess the caring competence of family caregivers and provide proper suicide care education. In this perspective, caregiver could be involved systematically at the time of discharge of patients at suicide risk (Sun et al., 2014).

Caregivers and healthcare professionals should strive to create between themselves and with the suicidal patient a back-and-forth dynamic, wherein the risk of caregiver burden is a constant threat and urges caution on the part of all involved.

Tacitly taken for granted, the role of caregivers is at best reduced to that of auxiliaries, providing little more than emotional or material support to the patient, for whom they may as a last resort act as a spokesperson (Fredman and Daly, 1993). The patient will surely benefit from the implication of his/her caregiver in a preventative approach.

## Toward the Involvement of Caregivers in Suicide Prevention Strategies?

Few studies have emphasized the family caregivers of suicidal individuals. No study has explored the relationship between family caregivers' caring stress with suicidal attitudes and suicide care ability. What does the caregiver paper adds to existing knowledge (Chiang et al., 2015)? Caregivers may be the primary interface with the health care system often receive inadequate support from health professionals and frequently feel abandoned and unrecognized by the health care system.

Mental health clinicians could help caregivers become aware of the emotional pain that suicidal people experience and then promote their positive attitudes toward their suicidal relatives. Caregivers could increase their ability to care for their suicidal relatives, which could reduce the numbers of suicides. Caregivers naturally play an essential role in supporting the well-being and care of patient at risk of suicide. Clinicians should identify their patients' caregivers, inquire about their caregiving experience, and benefit from a caregiver assessment. They should engage caregivers as proactive partners in care based on the involvement they can handle and the help they may need. We advocate that, as other preventative interventions, the efficacy of the involvement of caregivers in a suicide prevention strategy should be assessed in further researches.

## Author Contributions

All authors listed have made a substantial, direct and intellectual contribution to the work, and approved it for publication.

### Conflict of Interest Statement

The authors declare that the research was conducted in the absence of any commercial or financial relationships that could be construed as a potential conflict of interest.
